# A case of fulminant respiratory diphtheria in a 24-year-old Afghan refugee in Austria in May 2022: a case report

**DOI:** 10.1007/s15010-022-01926-4

**Published:** 2022-09-30

**Authors:** M. T. Traugott, S. Pleininger, S. Inschlag-Tisch, B. Eder, T. Seitz, A. Merrelaar, J. Reiß-Kornfehl, J. Fussi, S. Schindler, M. Blaschitz, F. Heger, A. Indra, M. Karolyi, M. Staudacher, T. Oelschlaegel, W. Hoepler, S. Neuhold, C. Wenisch

**Affiliations:** 1 IV Medical Department with Infectious Diseases and Tropical Medicine, Clinic Favoriten, Kundratstraße 3, 1100 Vienna, Austria; 2 Austrian Reference Centre for Diphtheria, Austrian Agency for Health and Food Safety, Vienna, Austria; 3 Department of Otorhinolaryngology, Hospital of Wiener Neustadt, Wiener Neustadt, Austria; 4 Department of Internal Medicine, Cardiology and Nephrology, Hospital of Wiener Neustadt, Wiener Neustadt, Austria

**Keywords:** Toxin-producing *Corynebacterium diphtheriae*, Respiratory diphtheria, Austria, Afghan, Migration, Diphtheria antitoxin

## Abstract

**Purpose:**

Raising awareness of respiratory diphtheria and for the importance of early antitoxin administration.

**Methods:**

Report of a case of fulminant, imported respiratory diphtheria in an otherwise healthy 24-year-old Afghan refugee in Austria in May 2022.

**Result:**

This was the first case of respiratory diphtheria in Austria since 1993. Diphtheria antitoxin was administered at an already progressed disease stage. This delay contributed to a fulminant disease course with multiorgan failure and death.

**Conclusion:**

In high-income countries with low case numbers, awareness of respiratory diphtheria and for the importance of early antitoxin administration must be raised.

## Introduction

Since the development of the diphtheria antitoxin in 1890 [1] as well as the diphtheria toxoid vaccine in the early 1920s [2], cases of respiratory diphtheria became rare in Europe. The incidence in the WHO European region has declined until 2011. In the last decade the number of cases undulated between 32 and 73 cases per year of respiratory and cutaneous diphtheria [3]. The present case has been the first case of respiratory diphtheria in Austria for the last 29 years [4].

## Case report

**Fig. 1:**
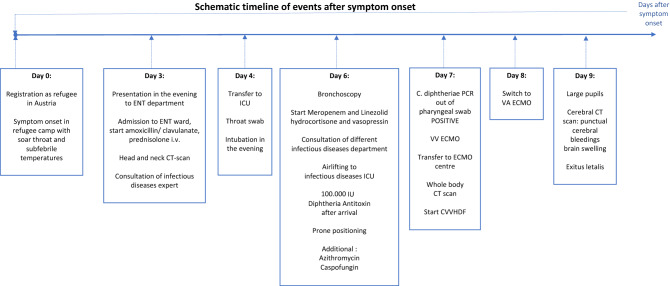
timeline of disease progression. *ENT* ear nose and throat, *CT* computed tomography, *ICU* intensive care unit, *IU* international units, *C.* Corynebacterium, *PCR* polymerase chain reaction, *VV ECMO* veno-venous extracorporeal membrane oxygenation, *CVVHDF* continuous veno-venous hemodiafiltration, *VA ECMO* veno-arterial extracorporeal membrane oxygenation

**Fig. 2:**
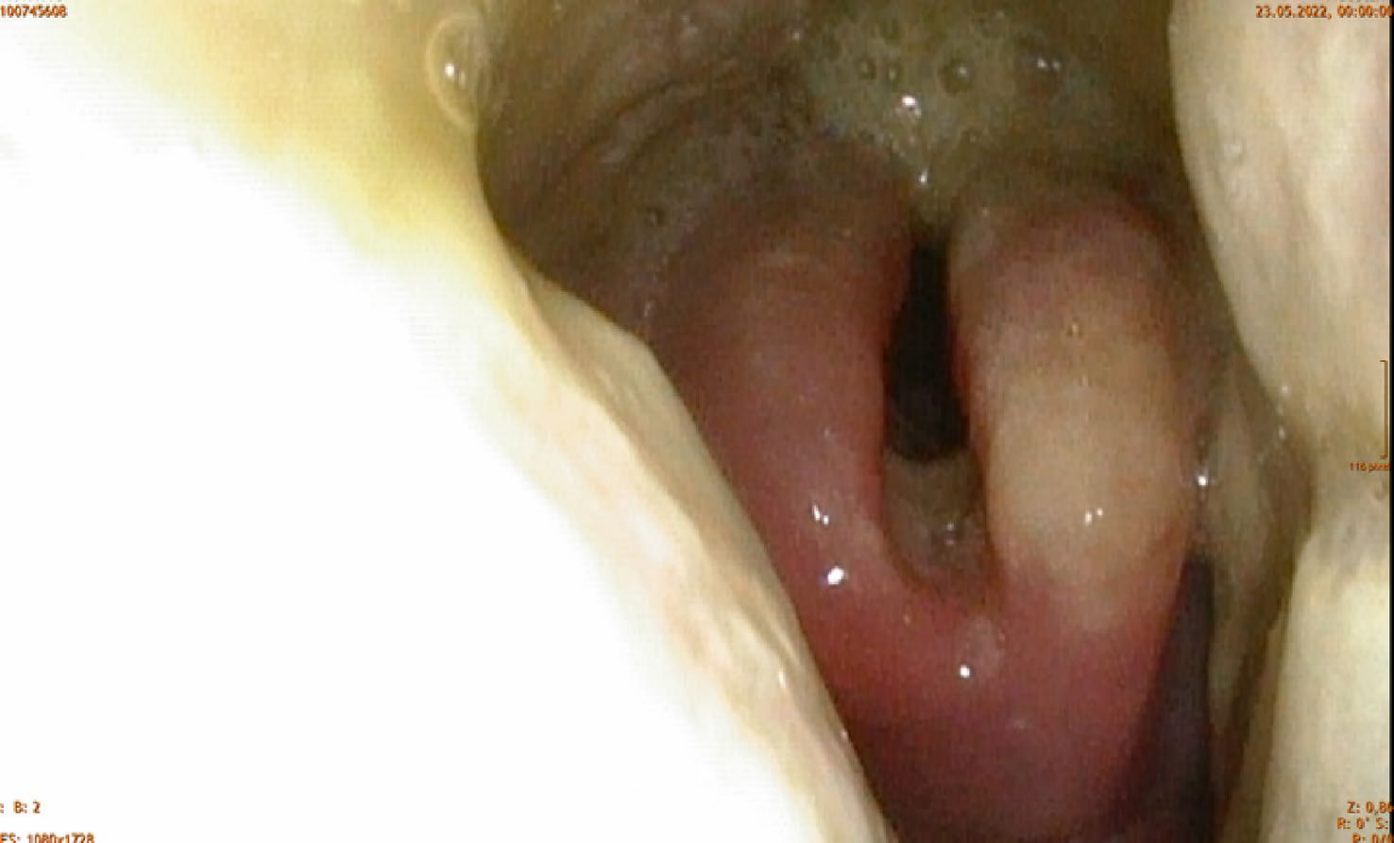
Flexible endoscopy with membranes in the hypopharynx and edematous epiglottis (day 3) (© J. Reiß-Kornfehl)

On 23rd May 2022, a 24-year-old Afghan refugee accommodated in an Austrian refugee center presented to the ear nose, and throat (ENT) department of the hospital of Wiener Neustadt. The patient reported a massive sore throat and a slightly elevated temperature for four days (See Fig. 1). Clinical examination showed kissing tonsils covered with membranes with the left tonsil partly destroyed. Flexible endoscopy (See Fig. 2) revealed purulent secretion and thick membranes on the whole pharyngeal wall. The neck lymph nodes were bilaterally swollen. An empiric intravenous (i.v.) antibiotic therapy with amoxicillin/clavulanate (2.2 g every 8 h) was started. In addition, 500 mg prednisolone i.v. were administered combined with adrenaline inhalations and analgetic therapy. A peritonsillar abscess was ruled out by computed tomography (CT). An infectious diseases expert was consulted by telephone. The case was described in detail, and the continuation of amoxicillin/clavulanate was recommended.

Because of increasing constriction of the airway, intubation had to be performed the next day. Over the next hours oxygenation worsened and resulted in acute respiratory distress syndrome (ARDS). On day 6 after symptom onset, the calculated antibiotic therapy was escalated to meropenem (1 g i.v. every 8 h) and linezolid (600 mg every 12 h) because of markedly rising inflammation markers (see Table 1). Vasopressors had to be started due to hemodynamic instability. Acute kidney failure developed. Due to the rapid onset of multi-organ failure and persistent suspicion of diphtheria, a different infectious diseases department was contacted.

**Table 1:** Laboratory parameters on the days after symptom onset

Days after symptom onset	Day 3	Day 4	Day 5	Day 6 a.m	Day 6 p.m	Day 7 a.m	Day 7 p.m	Day 8 (worst values)	Day 9 (worst values)
Leukocytes (3.9–10.2 G/l)	21	16	44	50	61	73	81	35	27
Thrombocytes (150–370 G/l)	173	134	112	58	41	32	25	27^a^	25^a^
CRP (< 5 mg/l)	180	167	210	240	258	209	165	64	58
PCT (< 0.5 ng/ml)		0.5	0.9	1.9					
IL-6 (< 7 pg/ml)		48	490	168		164	1077	1979	854
INR	< 1.2	1.3	1.4	1.3	1.18	1.17	1.22	3.1^a^	2.4^a^
aPTT (26–38 s)	38	40	39	44	41	41	44	91^a^	62^a^
Haptoglobin (0.3–2 g/l)							2.58		
Fibrinogen (1.8–3.5 g/l)					5.8	5.6	5.0	1.5^a^	1.8^a^
Creatinine (0.6–1.1 mg/dl)	0.9	0.7	1.0	2.9	3.7	4.95	5.51	4.86	2.51
GFR (> 90 ml/min/1,73 m^2^)	114	129	112	29	21	15	13	CVVHDF	CVVHDF
GOT (< 50 U/l)	29	25	28	67	81	83	86	8440	13,460
GPT (< 50 U/l)	28	24	30	49	45	41	39	2540	3551
LDH (< 250 U/l)	268				940	1000	1200	10,800	134,600
Albumin (35–52 G/l)		39	31	25		20	13	25^a^	30^a^
Arterenol rate (µg/kg/min)			0.13	0.13	0.04	0.22	0.9	1.8	0.8
paO2/FiO2 ratio (> 350 mmHg)			145	150	80	120	60	VV ECMO	VA ECMO

*CRP* C-reactive protein, *PCT* procalcitonin, *IL-6* Interleukine-6, *INR* international normalized ratio, *aPTT* activated prothrombin time, *GFR* glomerular filtration rate, *GOT* glutamate oxalacetate transaminase, *GPT* glutamate pyruvate transaminase, *LDH* lactate dehydrogenase, *G* giga, *L* liter, *g* gram, *U* units, *ng* nanogram, *ml *milliliter, *pg* pictogram, *µg* microgram, *kg* kilogram, *min* minute, *paO2/FiO2* partial arterial oxygen/fraction of inhaled oxygen, *VV ECMO* veno-venous extracorporeal membrane oxygenation, *VA ECMO *veno-arterial extracorporeal membrane oxygenation, *mmHg* millimeter of mercury, *CVVHDF* continuous veno-venous hemodiafiltration

^a^After substitution

Subsequently, the patient was airlifted to Clinic Favoriten, which is the only treatment center in Austria storing diphtheria antitoxin. There, 100.000 I.U. diphtheria-antitoxin were administered i.v. following the instructions of the manufacturer. In the consecutive bronchoscopy the full extent of the membranes, reaching all the way to the lung periphery, became visible. Inflammation markers increased further, the platelet count decreased, and acute kidney injury persisted. The antibiotic therapy was escalated with additional azithromycin (500 mg i.v. once daily) and caspofungin. Due to severe ARDS, the patient was turned into prone position. Catecholamine demand was increasing, and the patient showed bleeding stigmata in the mouth and nose.

On day 7 after symptom onset *C. diphtheriae* was confirmed in the throat swab and toxin-gene polymerase chain reaction (PCR) was positive for subunits A and B. On the same day the patient desaturated down to 85% saturation of peripheral oxygen (SpO2). After ruling out all reversible causes for hypoxemia an ECMO center was contacted for the implantation of a veno-venous extracorporeal membrane oxygenation (VV ECMO (1)). Because of the patient’s respiratory instability, the implantation was performed by an ECMO retrieval team on site.

After the oxygenation had been restored, the patient was referred to a specialized ECMO-ICU. There, he presented with hyperdynamic septic shock, ARDS, acute kidney failure, and disseminated intravascular coagulation (DIC). Shock management consisted of high-frequent dynamic assessment of fluid responsiveness, large volumes of crystalloid and albumin infusions, high-dose combination vasopressor therapy including norepinephrine, epinephrine, and argipressin, as well as hydrocortisone i.v. Continuous veno-venous hemodiafiltration was started and, in the context of diffuse mucosal bleeding, DIC management required the administration of clotting factors and thrombocyte concentrates. Accordingly, ECMO was administered without any anticoagulation therapy throughout the course. On day 8, the patient developed additional ischemic hepatitis and, ultimately, septic cardiomyopathy with resulting hypodynamic septic shock. Thus, VV ECMO had to be switched to venoarterial ECMO [5] after a trial of dobutamine had failed. On day 9, the patient presented with wide, light rigid pupils. A cerebral CT scan revealed intracerebral spot bleedings and cerebral swelling. The young man died 9 days after the symptom onset of respiratory diphtheria.

## Microbiology and molecular investigation

Both pharyngeal samples yielded growth of *Corynebacterium diphtheriae* var mitis. PCR for diphtheria toxin subunits A and B [6] was positive, and toxin production was confirmed by ELEK-test. Minimum inhibitory concentrations (MICs) of antimicrobials were determined by Etest (bioMerieux SA, Marcy l’Etoile, France), in accordance with the manufacturer’s instructions. The MICs of ten antimicrobials and where available, interpretations according to EUCAST version 12.0 [7] and CLSI M45 2015 guideline [8], are shown in Table 2. One of the pharyngeal isolates was sequenced with Next Seq 2000 (Illumina, San Diego, CA, USA), with 151 bp paired-end reads. *C. diphtheriae* sequences were de novo assembled using SPAdes algorithm and further analyzed with Seqsphere + client version 8.3.4 (Ridom, Muenster, Germany). A core genome multilocus sequence type (cgMLST) comprising 1494 targets an accessory genome comprising 660 targets were created with one seed genome and 35 query genomes, thereof 13 complete genomes (bold), 1 chromosome, and 21 whole genome shotgun sequencing projects. (NCBI accession numbers: reference genome: *NC_002935.2, query Genomes (35): *NC_016782.1, *NC_016799.1, *NC_016800.1, *NC_016801.1, *NC_016785.1 *NC_016786.1, *NC_016802.1, *NC_016787.1, *NC_016788.1, *NC_016783.1, *NC_016789.1, *NC_016790.1, *NZ_CP018331.1, **NZ_LN831026.1 chromosome 1, MIOA00000000.1, *MINY00000000.1, *MINZ00000000.1, *MIOB00000000.1, *MIYN00000000.1, *MIOG00000000.1, *MIOJ00000000.1, *MIOK00000000.1, *MIOC00000000.1, *MIOD00000000.1, *MIOE00000000.1, *MIOL00000000.1, *MIOM00000000.1, *MIYO00000000.1, *MIYQ00000000.1, *MION00000000.1, *MIYS00000000.1, *MIYP00000000.1, *MIOO00000000.1, *MIOP00000000.1 and *MIOR00000000.1.).

**Table 2:** Antimicrobial minimum inhibitory concentration of *Corynebacterium diphtheriae* var mitis causing lethal respiratory diphtheria, Austria, May 2022 (*n* = 1 strain)

Antimicrobial	Mic in mg/l	interpretation (EUCAST v 12.0, CLSI M45 2015 guideline) (a, b)
Penicillin g	0.125	Susecptible (CLSI)
Clindamycin	0.75	Resistant (EUCAST)
Erythromycin	0.064	Susceptible (CLSI)
Azithromycin	1	N.A
Rifampicin	< 0.002	Susceptible (EUCAST)
Vancomycin	1	Susceptible (EUCAST and CLSI)
Ciprofloxacin	0.064	Susceptible, increased exposure (EUCAST) susceptible (CLSI)
Linezolid	0.25	Susceptible (EUCAST)
Tetracyclin	0.125	Susceptible (EUCAST)
Meropenem	0.032	Susceptible (CLSI)

*EUCAST* European committee on antimicrobial susceptibility testing, *CLSI* clinical and laboratory standards institute, *MIC* minimum inhibitory concentration, *NA* not applicable (due to lack of interpretative breakpoints)

^a^The European Committee on Antimicrobial Susceptibility Testing. Breakpoint tables for interpretation of MICs and zone diameters. 2022;Version 12.0

^b^CLSI. Methods for Antimicrobial Dilution and Disk Susceptibility Testing of Infrequently Isolated or Fastidious Bacteria. CLSI guideline M45, Wayne, PA: Clinical and Laboratory Standards Institute. 2015

The isolate was assigned to sequence type (ST) 574 with the MLST profile atpA: 2, dnaE: 10, danK: 3, fusA: 1, leuA: 3, odhA: 3, rpoB: 2 and was confirmed to carry the diphtheria toxin gene.

### Infection control

The case of respiratory diphtheria was reported to the Austrian health authority, which started contact tracing: the patient had first been registered in Austria on 20th May 2022, the day of symptom onset. He had shared a room with three other asylum seekers, who were all untraceable.

## Discussion

In Austria, no infections with *C. diphtheriae* have been detected from 1993 until 2014, when a case of cutaneous diphtheria was diagnosed in a teenager from Eastern Africa [4]. Since then, occasional infections or colonization with toxigenic *C. diphtheriae* and *ulcerans* have been reported. Up to the present case, none of these infections have shown a toxic disease course [9].

In Europe, only rare cases of respiratory diphtheria have been recently reported: A 6-year old in Spain survived the infection in 2015 [10]. Respiratory diphtheria was lethal for an unvaccinated child in Belgium in 2016 [11]. In 2018 two cases have been reported in Spain and Latvia [12].

In Afghanistan a diphtheria outbreak was reported in 2002 with 854 reported cases (40.7 incidence rate (IR)). Subsequently, the incidence had declined down to zero reported cases. Since 2018 cases have been rising again to 61 in the year 2020 (1.5 IR) [13]. Globally, the diphtheria incidence is currently declining with 8638 reported cases in 2021 (1.3 IR) after a peak of 22,986 cases in the year 2019 (3.4 IR). Most of these cases in 2019 have been reported in the WHO African region (17.4 IR) followed by the WHO South-East Asian region (5.1 IR) [13]. These data include respiratory and cutaneous diphtheria [14].

The administration of diphtheria antitoxin is crucial for the successful treatment of respiratory diphtheria [15]. It must be given as soon as possible, ideally at once when diphtheria is suspected [16], and must not be delayed to wait for laboratory confirmation [16]. The antitoxin consists of neutralizing antibodies that can only bind the diphtheria toxin before cell entry [17]. In the cell, the toxin irreversibly causes cell death by inactivating elongation factor 2 [18]. Antibiotics have no effect on the circulating diphtheria toxin and are therefore insufficient as treatment [16], but they can inhibit the production of more diphtheria toxin, and they effectively prevent disease transmission [16]. The initial empirical antibiotic therapy with amoxicillin clavulanate in the present case was chosen because the infectious diseases expert mainly suspected bacterial tonsillitis in the patient’s very early disease course with the differential diagnoses of angina Plaut Vincenti or Lemierre’s syndrome.

Various antibiotics can be used for diphtheria treatment including penicillin, erythromycin and other macrolide antibiotics, clindamycin as well as linezolid and vancomycin in resistant strains. The bactericidal penicillin or the bacteriostatic erythromycin are recommended as first line therapy [19]. A small study comparing penicillin and erythromycin found a shorter time to fever clearance in the penicillin group, but also one patient with treatment failure and one with relapse. 27% of the isolates were resistant to erythromycin [20].

In 2020, 12 strains of *C. diphtheriae* and *ulcerans* have been detected in Austria from human and animal samples. None of the samples caused respiratory infections. Using the breakpoints of the European Committee of Antimicrobial Susceptibility testing [7], all isolates were sensible for linezolid and rifampicin. Three isolates (23%) were resistant to clindamycin, five (38%) were resistant to ciprofloxacin and five (38%) to penicillin [21].

The authors found no data supporting the use of protein synthesis inhibiting antibiotics such as linezolid and clindamycin in diphtheria. No evidence exists for combined antibiotic treatment. Because of reports of antimicrobial resistances to erythromycin [20, 22, 23], penicillin [23, 24], clindamycin [25] and multidrug resistance in 10.4% [23], combined antibiotic therapy is probably reasonable until antimicrobial resistance testing has been performed. Clindamycin resistance is especially frequent in *C. ulcerans* [26]. The broad antibiotic therapy in the present case was chosen to empirically cover also other causes of ARDS and multiorgan failure in the context of a life-threatening infection.

The team was notified about the positive result of the diphtheria toxin PCR four days after the first hospital contact, when the swab was taken. This delay was partly caused by a public holiday in Austria when microbiological laboratories are not working. Furthermore, the sample had to be transported 60 km from the rural hospital in Wiener Neustadt to the diphtheria reference center in Vienna where the toxin PCR is available.

In the present case, antitoxin was administered at a progressed stage of the disease course. This delay might be explained by a lack of knowledge on antitoxin availability as well as the loss of familiarity with this rare disease. Information about antitoxin availability is therefore crucial and must be shared with health care providers to prevent any delay of antitoxin administration in unvaccinated patients with respiratory diphtheria [27]. Immuno-absorption therapy was not used because of a lack of scientific evidence for immune-absorption therapy. Furthermore, a positive effect was not expected by the treating physicians because the circulating antitoxin was neutralized by the antitoxin, and the intracellular toxin was not available for immuno-absorption.

The diphtheria toxoid vaccination protects with an efficacy of 98% against toxic diphtheria disease [2]. Poor health care systems, vaccine skepticism, religious beliefs, fundamentalism, and lack of awareness because of the rarity of the disease might all be factors leading to insufficient vaccine coverage in low [28] and highly developed [29, 30] countries. The seroprevalence of diphtheria antibodies is decreasing with age because of low booster vaccination rates [29, 30]. Diphtheria toxoid vaccine coverage of children in Austria used to be about 90%. Disturbingly, a decrease down to 85% full vaccine coverage has been reported since 2018 [31].

A study, that was carried out in 2013 in Afghanistan, found that 31% of children were only partially vaccinated according to the Afghan immunization plan, and 18% were not vaccinated at all. The vaccine coverage rate ranged grossly between Afghan provinces from 2.5% to 83% [32]. After 2013 immunization programs have been reinforced, improving the diphtheria toxoid vaccine coverage in children to a peak of 72% in 2018/2019 [33]. Since then, vaccine coverage in children has been declining, presumably because of political instability and the Taliban’s seizure of power.

Due to insufficient vaccination coverage and ongoing strong migration movements, further cases of respiratory diphtheria must be expected in Austria and other European countries. Awareness of the importance of early antitoxin administration is the key to prevent further lethal cases.

## Data Availability

More data is available by contacting the corresponding author.
