# Correction: A case of fulminant respiratory diphtheria in a 24-year-old Afghan refugee in Austria in May 2022: a case report

**DOI:** 10.1007/s15010-022-01964-y

**Published:** 2023-01-13

**Authors:** M. T. Traugott, S. Pleininger, S. Inschlag-Tisch, B. Eder, T. Seitz, A. Merrelaar, J. Reiß-Kornfehl, J. Fussi, S. Schindler, M. Blaschitz, F. Heger, A. Indra, M. Karolyi, M. Staudacher, T. Oelschlaegel, W. Hoepler, S. Neuhold, C. Wenisch

**Affiliations:** 1 IV Medical Department with Infectious Diseases and Tropical Medicine, Clinic Favoriten, Kundratstraße 3, 1100 Vienna, Austria; 2 Austrian Reference Centre for Diphtheria, Austrian Agency for Health and Food Safety, Vienna, Austria; 3 Department of Otorhinolaryngology, Hospital of Wiener Neustadt, Wiener Neustadt, Austria; 4 Department of Internal Medicine, Cardiology and Nephrology, Hospital of Wiener Neustadt, Wiener Neustadt, Austria

**Correction: Infection** 10.1007/s15010-022-01926-4

Figure 1 in the original version of this article has been replaced.

**Fig. 1:**
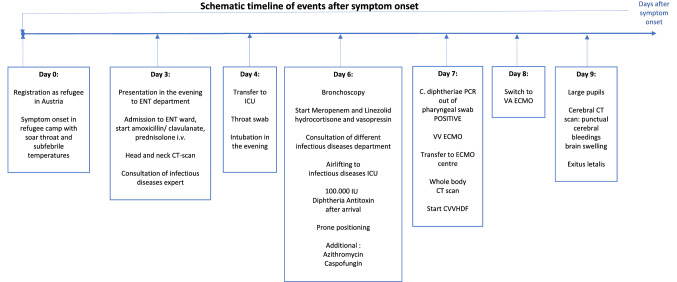
Timeline of disease progression. *ENT* ear nose and throat, *CT* computed tomography, *ICU* intensive care unit, *IU* international units, *C.* Corynebacterium, *PCR* polymerase chain reaction, *VV ECMO* veno-venous extracorporeal membrane oxygenation, *CVVHDF* continuous veno-venous hemodiafiltration, *VA ECMO* veno-arterial extracorporeal membrane oxygenation

The original article has been corrected.

